# The Use of Flexible Rubber in the Retention of Artificial Dentures after Other Means have Failed

**Published:** 1907-06-15

**Authors:** Newell H. Grove

**Affiliations:** Wyoming, Ohio


### The Use of Flexible Rubber in the Retention of Artificial Dentures after Other Means have Failed.

BY NEWELL H. GROVE, WYOMING, OHIO.

My clinic consists in the demonstration of a useful attachment for artificial vulcanite dentures, both upper and lower, whereby advantage of the atmospheric pressure and adhesion of contact is taken in such a manner as to hold the dentures firmly in the mouth of the patient so that they will not dislodge or become loosened during the process of mastication or during conversation.

This method is especially applicable where, after you have made a denture, you find that owing to the extreme flatness and hardness of the tissues, it will not stay in place. Then apply the strip of flexible rubber to the denture, and it will adhere so firmly that quite an effort will be necessary to dislodge it.

The process of attachment of said flexible strip is as follows: The denture, either upper or lower, is made and vulcanized in the usual manner, and the edges of the plate trimmed so as to completely free the muscles when the mouth is open or closed, and in case of lower allowing for free movement of the tongue. Then a ledge $\frac{1}{8}$ inch, or slightly less in width, is cut with a fissure bur to the depth of the thickness of the flexible rubber to be used. This ledge or groove is made completely around the rim or edge of the plate on the side next to the gums and across the back of the plate, so that it is continuous around the whole plate.

The bottom of this ledge or groove is painted or coated over with a solution of base-plate rubber in chloroform, and allowed to dry. Then cut a strip of Doherty's flexible or palate rubber, 3-16 of an inch, or slightly more in width, and place it carefully in this ledge and pat it down tightly and smoothly with a spatula, the fingers or other instrument, leaving the flexible rubber projecting beyond the edge of the plate. The ends of the piece or pieces of flexible rubber are thoroughly united by means of a warm spatula, so that it forms a continuous band or piece around the plate.

The whole is then invested in plaster in flask and revulcanized, after which any necessary trimming and polishing may be done.

